# Molecular mechanisms underlying psoriasis and depression: an integrated analysis using mendelian randomization, transcriptomics, and single-cell sequencing

**DOI:** 10.3389/fmmed.2026.1770665

**Published:** 2026-04-15

**Authors:** Baojun De, Wenfeng Bao, Nagongbilige Hea, Jun Fang

**Affiliations:** 1 Department of Psychosomatic Medicine, Inner Mongolia Traditional Chinese and Mongolian Medical Research Institute, Hohhot, Inner Mongolia, China; 2 Mongolian Psychosomatic Medicine Department, Inner Mongolia Ajitai Mongolian Hospital, Ordos, Inner Mongolia, China

**Keywords:** comorbidity, depression, ferroptosis, folic acid, immune cells infiltration, mendelian randomization, multi-omics integration, psoriasis

## Abstract

**Background:**

Psoriasis, an immune-mediated systemic inflammatory disease affecting skin, vessels, and joints, often co-occurs with depression. Routine depression screening is vital, as mood disorders link to inflammation, visible lesions, and functional limitations.

**Methods:**

The study integrated Mendelian randomization (MR), transcriptomics, and single-cell omics via public databases to explore comorbidity mechanisms.

**Results:**

MR identified 340 psoriasis-related and 307 depression-related eQTL-gene associations; 9 intersected. LASSO found 4 key Genes (*MAP3K20, WARS2, TBXAS1, ABHD15*), enriched in IL-17/NF-κB/FoxO pathways, cholesterol metabolism, and synaptic cycling. They correlated with immune infiltration, ferroptosis, and specific cell localization. Folic acid (from CTD) targeted 3 genes.

**Conclusion:**

These 4 genes mediate comorbidity via inflammation, immune metabolism, and ferroptosis. Folic acid pathways have therapeutic value, laying a foundation for precision therapy.

## Graphical Abstract

**Figure:**
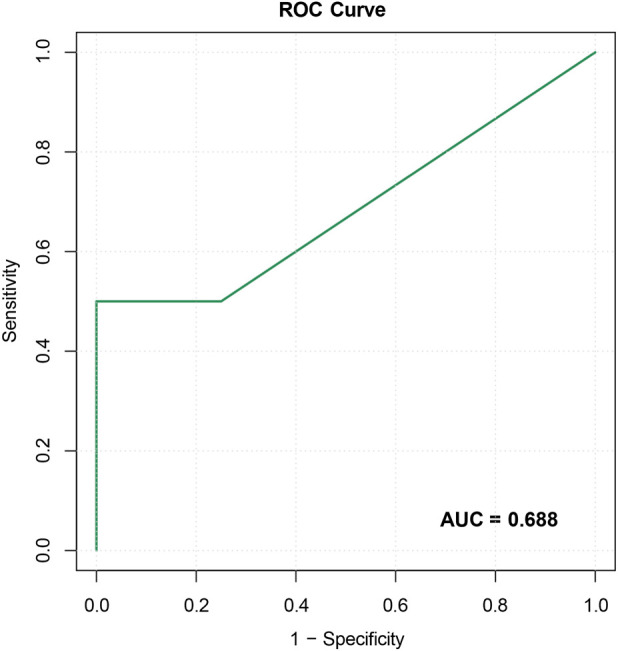

## 1 Introduction

Psoriasis is a chronic immune-mediated inflammatory disease affecting 2%–3% of the global population (∼125 million people) (National Psoriasis Foundation, 2022; Michalek et al., 2016). Pathologically, it features keratinocyte hyperproliferation, differentiation disorders, vascular changes, and immune imbalance centered on the IL-23/Th17 axis (Lowes et al., 2014). The core pathway involves IL-23-secreting dendritic cells activating Th17 cells to produce IL-17 cytokines (Di et al., 2009), inducing keratinocyte chemokine/antimicrobial peptide production and driving proliferation dysregulation and immune amplification (Alecu et al., 2020). Neutrophils migrate to skin, accumulate in stratum corneum forming micropustules, and generate NETs that enhance dendritic cell-Th17 interactions and IL-17 inflammation (Lin et al., 2011; Herster et al., 2020).

Psoriatic inflammation extends systemically, associating with psoriatic arthritis and elevated cardiovascular risk (Ritchlin et al., 2017; Armstrong et al., 2013). Risk factors include genetic susceptibility, environmental triggers (infection, trauma, climate, medications), immune dysregulation (Th17/Treg imbalance), metabolic abnormalities, lifestyle factors, and psychological stress, with comorbidities including cardiovascular disease, metabolic syndrome, and inflammatory bowel disease (Takeshita et al., 2017). Recent studies highlight metabolic and neuropsychiatric comorbidities (Dowlatshahi et al., 2014). Approximately 20% of psoriasis patients experience clinical depression, linked through genetic, inflammatory, neuroimmune, and psychosocial mechanisms (Dowlatshahi et al., 2014; Jeon et al., 2017).

Mendelian randomization confirms bidirectional causal relationships between psoriasis and depression, with 5-HTT polymorphism promoting both via serotonin signaling (Liu et al., 2023). Psoriasis exhibits systemic low-grade inflammation: the IL-23/Th17 axis releases IL-17A/F and IL-23 (Li et al., 2025), while dendritic cells and myeloid cells secrete TNFα, IL-1β, and IL-6 (Vičić et al., 2021; Wang et al., 2020). Th17/ILC3-derived IL-17A/F and IL-22 drive keratinocyte proliferation, which reciprocally produces *CXCL1/2, CCL20, I*L-36γ, and antimicrobial peptides, forming self-perpetuating inflammation (Hawkes et al., 2017). In depression, peripheral IL-6 and TNF-α cross the blood-brain barrier inhibiting neurotransmitter synthesis (Marek-Jozefowicz et al., 2022). IL-1β activates the HPA axis, causing glucocorticoid resistance and monoamine depletion. HPA overactivation elevates cortisol, reducing *BDNF* and impairing hippocampal function, while inflammatory factors inhibit tryptophan hydroxylase, disrupting serotonin metabolism (Tohid et al., 2016; Kleyn et al., 2020).

Psoriasis and depression form a vicious cycle through inflammatory cross-talk (IL-6, TNF-α, IL-17), HPA axis dysregulation, and tryptophan metabolism disorders. Th17/Th1 activation and pro-inflammatory cytokines affect neurotransmitter metabolism, neuroplasticity, and microglial activation via peripheral-central pathways (Tang et al., 2025; Hołdrowicz and Żebrowska, 2025; Dowlati et al., 2010; Troubat et al., 2021). Elevated IL-6/TNF-α maintain keratinocyte proliferation and increase depression susceptibility by crossing the blood-brain barrier, reducing 5-HT synthesis and altering tryptophan-kynurenine pathways. Psychosocially, visible lesions cause stigma and chronic stress, while pruritus, pain, and sleep disorders amplify negative emotions synergistically with inflammatory signals (Szepietowski and Reich, 2016; Saçmacı and Gürel, 2019; Vucemilovic et al., 2020; Daudén et al., 2024). This comorbidity reflects a multi-layered network of genetic susceptibility, systemic inflammation, neuroimmune remodeling, and psychosocial stress.

Management of both conditions emphasizes multidimensional goals: controlling lesions/inflammation, improving quality of life, and preventing comorbidities, with regular assessment using PASI/BSA/DLQI for psoriasis and PHQ-9/HAM-D for depression (Xiao et al., 2019; Chat et al., 2022; Papp et al., 2022; Nast et al., 2017; National Institute for Health and Care Excellence, 2022). Treatment relies on pharmacotherapy, lifestyle modification, and psychosocial support. Comprehensive intervention targeting both conditions theoretically achieves optimal outcomes, but underlying mechanisms require clarification.

Bioinformatics and gene chip technology reveal molecular mechanisms through high-throughput data and machine learning. Mendelian randomization (MR) validates causal relationships by avoiding confounding factors (Guo et al., 2024; Wang et al., 2025; Kelly et al., 2021). However, systematic analysis of core molecular networks in psoriasis-depression comorbidity is lacking. This study integrates MR, machine learning, transcriptomics, and single-cell omics to explore association mechanisms, molecular pathways, and common genes, providing theoretical basis for clinical intervention and individualized treatment.

## 2 Methods

### 2.1 Data acquisition

All data in this study were obtained from the GEO public database (https://www.ncbi.nlm.nih.gov/geo/). We acquired the depression bulk transcriptome dataset GSE80655 (Platform GPL11154, comprising 139 samples with 70 controls and 69 patients) and the psoriasis bulk transcriptome dataset GSE166388 (Platform GPL570, comprising 8 samples with 4 controls and 4 patients). Additionally, we obtained single-cell transcriptome datasets for both conditions: GSE213982 for depression (38 samples; 18 controls and 20 patients) and GSE220116 for psoriasis (21 samples; 11 controls and 10 patients) (see Table 1). For Mendelian randomization analysis (Smith and Ebrahim, 2003), exposure data were sourced from the eQTLGen consortium (https://www.eqtlgen.org), a database dedicated to deciphering the genetic architecture of blood gene expression. The outcome genome-wide association study (GWAS) data, based on populations of European ancestry, were obtained from the FinnGen biobank for psoriasis (finngen_R12_L12_PSORIASIS, defined through ICD codes, including 12,760 cases and 482, 181 controls) and from the European Bioinformatics Institute (EBI) database for depression (ebi-a-GCST005902, including 113,769 cases and 208,811 controls).

**TABLE 1:** Public datasets used in this study.

Dataset ID	Data file type	GPL number	Disease type	Total samples (n)	Controls (n)	Cases (n)
GSE80655	Series matrix file	GPL11154	Depression	139	70	69
GSE166388	Series matrix file	GPL570	Psoriasis	8	4	4
GSE213982	Single-cell data file	​	Depression	38	18	20
GSE220116	Single-cell data file	​	Psoriasis	21	11	10

### 2.2 Mendelian randomization analysis

Genetic variant identifiers (IDs) for target outcome variables were selected from genome-wide association study (GWAS) databases to establish potential causal associations using summary statistics of exposures and outcomes. Single nucleotide polymorphisms (SNPs) significantly associated with exposure factors at genome-wide significance level (P < 5e-8) were selected as initial instrumental variables (IVs). Linkage disequilibrium (LD) between SNPs was assessed, and high-LD SNPs (r^2^ > 0.001) within 10,000 kb were removed, retaining independent IVs with low LD. Weak instrument bias was excluded using the criterion of F-statistic ≥10. Four statistical methods evaluated causal associations between exposure factors and psoriasis/depression: inverse-variance weighted (IVW), MR-Egger regression (assuming instrument strength independent of direct effect), weighted median (providing valid estimates when ≤50% of instruments are invalid), and weighted mode (offering stronger causal effect testing power with lower bias and Type I error). For single-SNP-driven causal relationships, the Wald ratio method estimated effect values. Leave-one-out sensitivity analysis determined robust causal effect estimates.

### 2.3 Sensitivity analysis

We employed Mendelian randomization (MR) leave-one-out sensitivity analysis to assess the robustness of causal effect estimates between exposure factors and psoriasis/depression risk. This method sequentially removed individual SNPs and recalculated pooled causal effect sizes using inverse-variance weighted (IVW) method (Hemani et al., 2018). Point estimates and 95% confidence intervals (95% CI) were calculated after each removal. Forest plots visualized effect estimates after removing each SNP compared to overall effect estimates. Additionally, MR-Egger intercept tests and MR-PRESSO analysis systematically evaluated potential horizontal pleiotropy and overall causal association robustness.

### 2.4 Machine learning analysis

Using datasets GSE80655 and GSE166388, we performed feature variable selection using Lasso regression models implemented through R package “glmnet” (Friedman et al., 2010). Sample group labels were converted to binary variables, and gene expression profile data were incorporated as predictor variable matrices. Lasso regression (Tibshirani, 1996) achieved feature selection by introducing L1 penalties in the loss function, compressing regression coefficients and reducing insignificant variables to zero. Optimal penalty coefficient λ was determined through 10-fold cross-validation, selecting features with non-zero regression coefficients as candidate diagnostic markers.

### 2.5 GSEA analysis

Based on key gene expression levels, samples were divided into high and low expression groups, and GSEA (Subramanian et al., 2005) analyzed pathway differences between groups. MSigDB v7.0 annotated gene sets served as background for pathway differential analysis. Significantly enriched gene sets (adjusted p < 0.05) were ranked based on Enrichment Score. GSEA demonstrates significant value in disease subtyping research.

### 2.6 Immune infiltration

CIBERSORT (Chen et al., 2018), a support vector regression-based deconvolution algorithm, analyzes gene expression matrices and evaluates immune cells composition. Its reference dataset contains 547 gene markers distinguishing 22 immune cells subtypes including T cells and B cells. We applied CIBERSORT to analyze sample data, inferring relative immune cells abundances and correlating gene expression levels with immune cells content.

### 2.7 Transcription factor regulatory network

We employed R package “RcisTarget” (Aibar et al., 2017) for transcription factor prediction based on motif analysis. NES depends on total motifs in the database. Beyond source data annotations, we supplemented annotations through similarity analysis and sequence alignment. For motif overexpression calculation, motif-gene set pair AUC was determined through sorted recovery curves. NES was derived from AUC distribution across all motifs.

### 2.8 Single-cell analysis

Using Seurat (Hao et al., 2021) package, we imported gene expression matrices and filtered cells based on UMI counts, detected genes, and mitochondrial/ribosomal gene percentages. Outliers were defined as three MADs from median: high UMI/gene counts indicated potential doublets; high mitochondrial/ribosomal percentages suggested apoptotic or fragmentary samples. DoubletFinder algorithm removed doublets. Data processing included normalization, cell cycle scoring, variable feature selection, and variance scaling using NormalizeData, CellCycleScoring, FindVariableFeatures, and ScaleData functions. PCA dimensionality reduction used RunPCA, with principal components selected by elbow criterion. Batch effects were corrected using Harmony algorithm, clustering similar cells in PCA space. RunUMAP performed non-linear dimensionality reduction, FindNeighbors constructed neighborhood graphs, and FindClusters performed cell clustering. Cell type annotation integrated CellMarker database, literature-based markers, and SingleR algorithm cross-validation.

### 2.9 CTD drug prediction

The Comparative Toxicogenomics Database (CTD) (Mattingly et al., 2003) integrates extensive interaction data between chemicals, genes, phenotypes, and diseases, supporting investigation of disease-related environmental exposures and potential drug mechanisms. The database contains over 46.64 million interaction records, covering over 2.3 million chemicals, 46,689 genes, 4,340 phenotypes, and 7,212 disease entries.

### 2.10 Molecular docking

For identified key Genes, we retrieved predicted three-dimensional protein structures through AlphaFold (Rives et al., 2021) database (https://alphafold.com/). Candidate drugs targeting these genes were predicted using Comparative Toxicogenomics Database (CTD; https://www.ctdbase.org/), obtaining compound and mechanism information. Chemical structure information was retrieved from PubChem database (https://pubchem.ncbi.nlm.nih.gov/). Molecular docking simulations used AutoDock software, with nine repetitions per ligand-receptor combination to assess binding stability. The lowest binding free energy conformation, excluding initial reference conformations, was analyzed. Final docking results were visualized using PyMOL molecular visualization system (version 2.5.2), displaying key binding sites and interaction patterns between ligand molecules and protein targets.

### 2.11 Statistical analysis

Mendelian randomization (MR) analysis requires three key assumptions: 1. relevance assumption - instrumental variables (IVs) must strongly correlate with exposure without direct outcome association; 2. independence assumption - IVs must be independent of all potential confounding factors; 3. exclusion restriction - IVs affect outcomes only through exposure. Potential bias must be evaluated when alternative pathways exist (e.g., horizontal pleiotropy). This study used R software (v 4.3.0) for MR analysis, employing two-sided tests with statistical significance at P < 0.05.

## 3 Results

### 3.1 Mendelian randomization analysis

**FIGURE 1:**
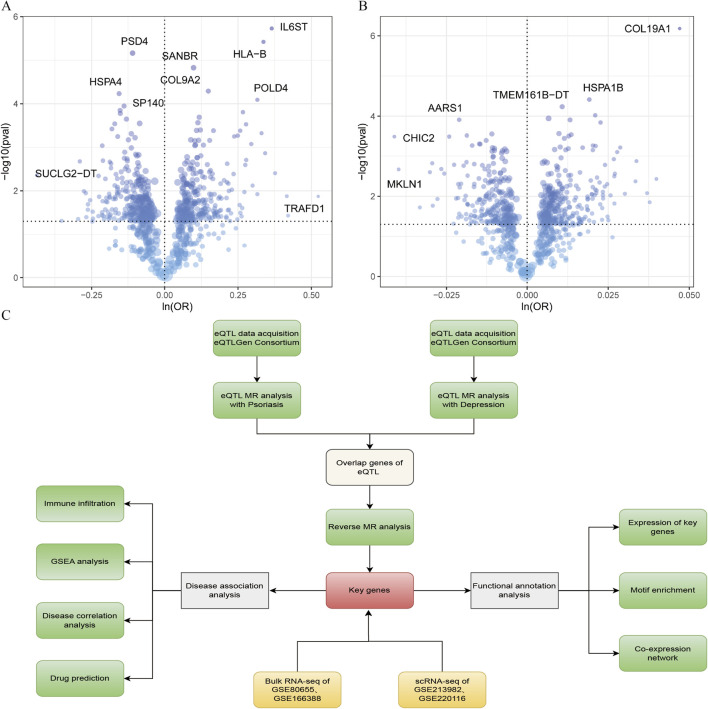
Volcano plots showing associations between genes and disease risk. **(A)** Gene associations with psoriasis risk. **(B)** Gene associations with depression risk. **(C)** Research flow chart.

The overall flow chart of this study is shown in Figure 1C. Based on summary statistics from 494,941 psoriasis-related samples (482, 181 controls and 12,760 cases) with outcome ID finngen_R12_L12_PSORIASIS, we employed extract_instruments and extract_outcome_data methods sequentially to identify 786 potential causal associations between eQTLs and outcomes. Further Mendelian randomization (MR) analysis revealed 340 significant causal relationships between genes and eQTL-positive outcomes (Supplementary Figure S1, inverse-variance weighted method IVW p-value<0.05). Results indicated that 176 genes, including *TYK2, UPB1, PREX1, MARK2*, and *PRKDC*, were potentially associated with reduced psoriasis risk (OR < 1), while 164 genes, including *F12, KLF6, HLA-B, IL6ST,* and *STK19B*, were associated with increased psoriasis risk (OR > 1). To validate the reliability of these 340 causal associations, we conducted sensitivity analyses. Leave-one-out analysis demonstrated that removing any single nucleotide polymorphism (SNP) did not significantly shift the overall effect estimate, suggesting the robustness of selected associations. The association strength between genes and psoriasis risk is visualized in a volcano plot (Figure 1A).

Using summary statistics from depression-related samples (outcome ID: ebi-a-GCST005902), we followed the same procedure and identified 721 potential causal associations between eQTLs and outcomes. MR analysis confirmed 307 significant causal relationships between genes and eQTL-positive outcomes (Supplementary Figure S2, IVW p-value < 0.05). Results showed that 127 genes, including *NOTCH4, PRDM11, MBD6, AARS1*, and *OPLAH*, were potentially associated with reduced depression risk (OR < 1), while 180 genes, including *PTAFR*, *ZNF610, ZNF525, BCL3*, and *CD74*, were associated with increased depression risk (OR > 1). Sensitivity analysis of the 307 gene causal associations demonstrated that removing any single SNP in leave-one-out testing did not significantly alter the overall effect estimate, confirming the robustness of selected associations. The association strength between genes and depression risk is visualized in a volcano plot (Figure 1B).

### 3.2 Identification of key genes and reverse mendelian randomization analysis

**FIGURE 2:**
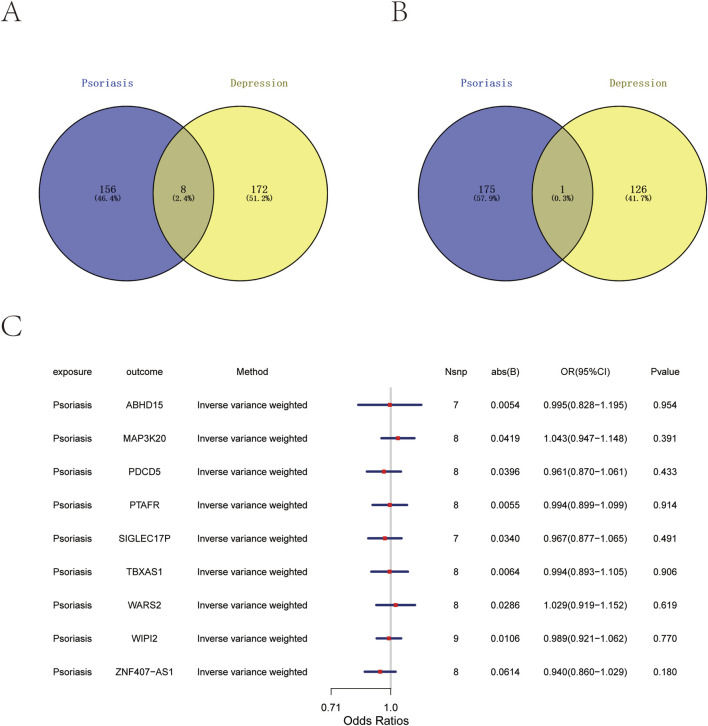
Shared genes between psoriasis and depression, and reverse Mendelian randomization analysis. **(A)** Venn diagram showing eight high-risk genes shared between psoriasis (blue) and depression (yellow). **(B)** Venn diagram showing one low-risk gene shared between psoriasis (blue) and depression (yellow). **(C)** Forest plot of reverse Mendelian randomization analysis (inverse variance weighted method) showing no significant causal effects of psoriasis on shared gene expression.

To identify key Genes affecting both psoriasis and depression, we intersect ed genes associated with high and low risk of both conditions using Venn diag rams. This analysis revealed one common gene associated with low disease ris k *(PDCD5*) and eight common genes associated with high disease risk (*WIPI2*, *WARS2*, *ABHD15*, *ZNF407-AS1*, *TBXAS1*, *MAP3K20*, *SIGLEC17P*, and *PTAFR*) (Figures 2A,B). In reverse MR analysis, we used finngen_R12_L12_PSORIAS IS and ebi-a-GCST005902 as exposure factors and selected the nine intersectio n genes as outcome data. The reverse MR analysis demonstrated that neither fi nngen_R12_L12_PSORIASIS nor ebi-a-GCST005902 showed causal relationship s with the eQTL levels of *WIPI2*, *WARS2*, *ABHD15*, *ZNF407-AS1*, *TBXAS1*, *M AP3K20*, *SIGLEC17P*, and *PTAFR* (Figure 2C).

### 3.3 Identification of key genes using machine learning

**FIGURE 3:**
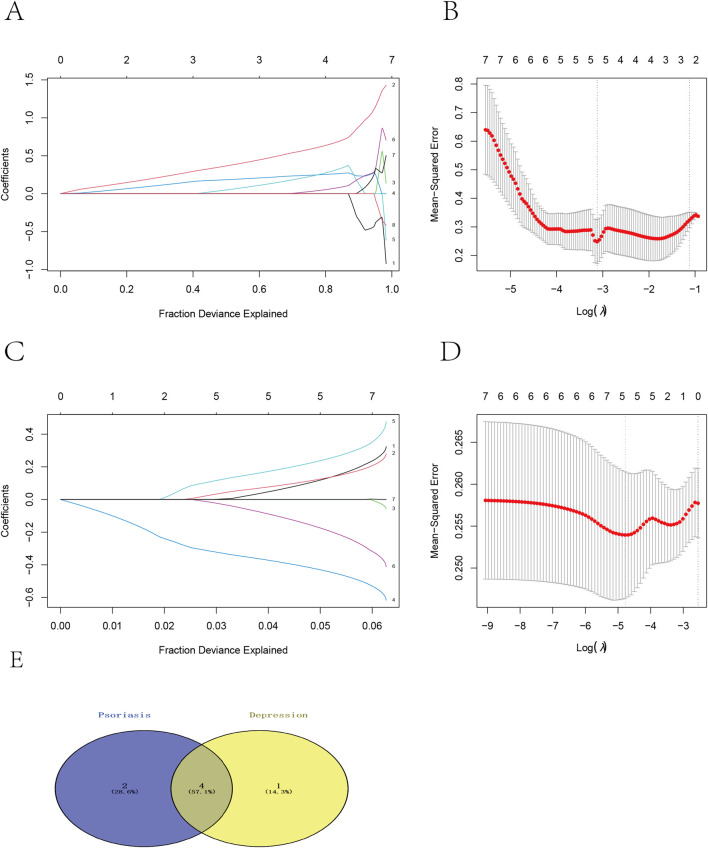
Feature selection of diagnostic markers for psoriasis and depression using Lasso logistic regression. **(A)** LASSO coefficient spectrum of characteristic genes for psoriasis. **(B)** Cross-validation for optimal penalty coefficient λ in psoriasis. **(C)** LASSO coefficient spectrum of characteristic genes for depression. **(D)** Cross-validation for optimal penalty coefficient λ in depression. **(E)** Four overlapping genes identified through machine learning screening between psoriasis and depression.

To further identify crucial genes influencing both psoriasis and depression, we employed Lasso logistic regression for feature selection of diagnostic markers using two datasets (GSE80655 and GSE166388), based on the aforementioned eight candidate genes. Using the “glmnet” package, we identified *MAP3K20*, *WARS2*, *PTAFR, TBXAS1, PDCD5*, and *ABHD15* as characteristic genes for psoriasis (Figures 3A,B), while *TBXAS1, MAP3K20, WARS2, WIPI2,* and *ABHD15* were identified as characteristic genes for depression (Figures 3C,D). Subsequently, we determined the common genes between the two conditions—*MAP3K20, WARS2, TBXAS1*, and *ABHD15*—as key Genes for further investigation of molecular mechanisms (Figure 3E).

### 3.4 GSEA analysis of key genes

**FIGURE 4:**
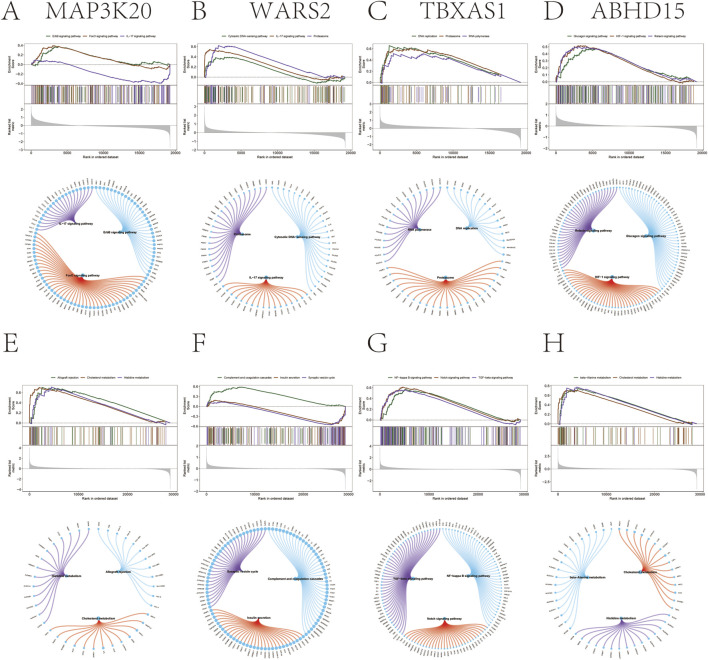
Gene Set Enrichment Analysis (GSEA) of key Genes in psoriasis and depression. **(A–D)** GSEA results for psoriasis showing enrichment of *MAP3K20* **(A)** in FoxO, IL-17, and ErbB signaling pathways; *WARS2* **(B)** in IL-17, proteasome, and cytosolic DNA-sensing pathways; *TBXAS1* **(C)** in DNA replication, proteasome, and RNA polymerase-related pathways; and *ABHD15* **(D)** in relaxin, HIF-1, and glucagon signaling pathways. **(E–H)** GSEA results for depression showing enrichment of *MAP3K20* **(E)** in cholesterol metabolism, allograft rejection, and histidine metabolism pathways; *WARS2* **(F)** in Synaptic vesicle cycle, insulin secretion, and complement and coagulation cascade pathways; *TBXAS1* **(G)** in TGF-β, NF-κB, and Notch signaling pathways; and *ABHD15* **(H)** in cholesterol metabolism, β-alanine metabolism, and histidine metabolism pathways.

Next, we investigated the specific signaling pathways associated with the key Genes to explore potential molecular mechanisms underlying disease progression. GSEA results for psoriasis revealed that *MAP3K20* was enriched in the FoxO signaling pathway, IL-17 signaling pathway, and ErbB signaling pathway (Figure 4A). *WARS2* was enriched in the IL-17 signaling pathway, proteasome, and cytosolic DNA-sensing pathway (Figure 4B). *TBXAS1* was enriched in DNA replication, proteasome, and RNA polymerase pathways (Figure 4C). *ABHD15* was enriched in the relaxin signaling pathway, HIF-1 signaling pathway, and glucagon signaling pathway (Figure 4D).

For depression, GSEA results showed that *MAP3K20* was enriched in cholesterol metabolism, allograft rejection, and histidine metabolism pathways (Figure 4E). *WARS2* was enriched in the Synaptic vesicle cycle, insulin secretion, and complement and coagulation cascades pathways (Figure 4F). *TBXAS1* was enriched in the TGF-beta signaling pathway, NF-kappa B signaling pathway, and Notch signaling pathway (Figure 4G). *ABHD15* was enriched in cholesterol metabolism, beta-alanine metabolism, and histidine metabolism pathways (Figure 4H).

### 3.5 Immune infiltration analysis

**FIGURE 5:**
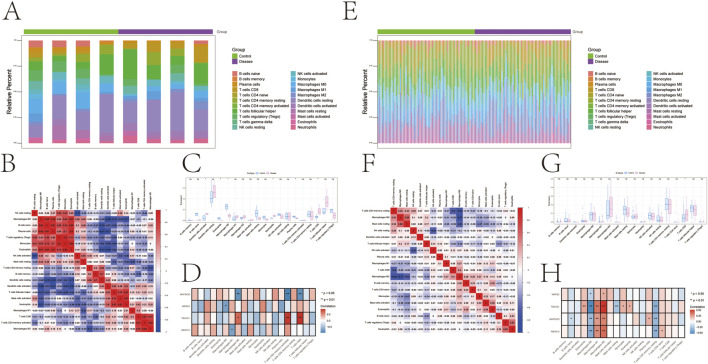
Immune infiltration analysis in psoriasis and depression. **(A)** Bar plot showing the proportion of immune cells distribution in psoriasis. **(B)** Heatmap of immune cells correlations in psoriasis. **(C)** Box plot of immune cells infiltration in psoriasis. **(D)** Heatmap showing correlations between key genes and immune cells in psoriasis. **(E)** Bar plot showing the proportion of immune cells distribution in depression. **(F)** Heatmap of immune cells correlations in depression. **(G)** Box plot of immune cells infiltration in depression. **(H)** Heatmap showing correlations between key genes and immune cells in depression. *P < 0.05, **P < 0.01, ***P < 0.001, ****P < 0.0001.

Using CIBERSORTx v1.0, we estimated the relative proportions of 22 immune cells types in the samples and visualized their distribution through box plots (Figures 5A,B). In psoriasis, compared to the control group, the disease group showed significantly elevated levels of activated dendritic cells, neutrophils, and CD8^+^ T cells, while exhibiting significantly decreased levels of eosinophils, M0 macrophages, and plasma cells (Figure 5C). Further investigation of relationships between key Genes and immune cells revealed significant negative correlations between *MAP3K20* and both CD8^+^ T cells and M2 macrophages; *WARS2* showed significant negative correlation with M0 macrophages; *TBXAS1* demonstrated significant positive correlations with CD8^+^ T cells and M2 macrophages; and *ABHD15* exhibited significant negative correlation with M1 macrophages (Figure 5D).

In the depression dataset, immune infiltration patterns and cellular correlations were visualized using stacked bar plots and correlation heatmaps (Figures 5E,F). The disease group similarly showed significant alterations in immune characteristics, particularly elevated CD8^+^ T cell levels (Figure 5G). Correlation analysis between key Genes and immune cells demonstrated that *ABHD15, MAP3K20, and TBXAS1* all showed significant positive correlations with M1 and M2 macrophages, while exhibiting significant negative correlations with M0 macrophages and resting memory CD4^+^ T cells. *WARS2* showed significant positive correlation with M2 macrophages and significant negative correlation with M0 macrophages (Figure 5H).

### 3.6 Analysis of key genes, disease-associated genes, and transcription factor regulatory networks

Disease-related genes for both psoriasis and depression were obtained from the GeneCards database (https://www.genecards.org/). We analyzed the expression differences of disease genes between groups, focusing on the top 20 genes ranked by relevance score that showed expression in the transcriptome. Significant differences were observed in the expression of *S100A7, KRT17, S100A7A, IL7R, IL2RA,* and *IRF2BP2* between psoriasis groups, while *PTCH1, BEST1,* and *DLG4* showed significant differences between depression groups. Furthermore, correlation analysis revealed significant associations between the expression levels of key Genes and disease-regulatory genes.

**FIGURE 6:**
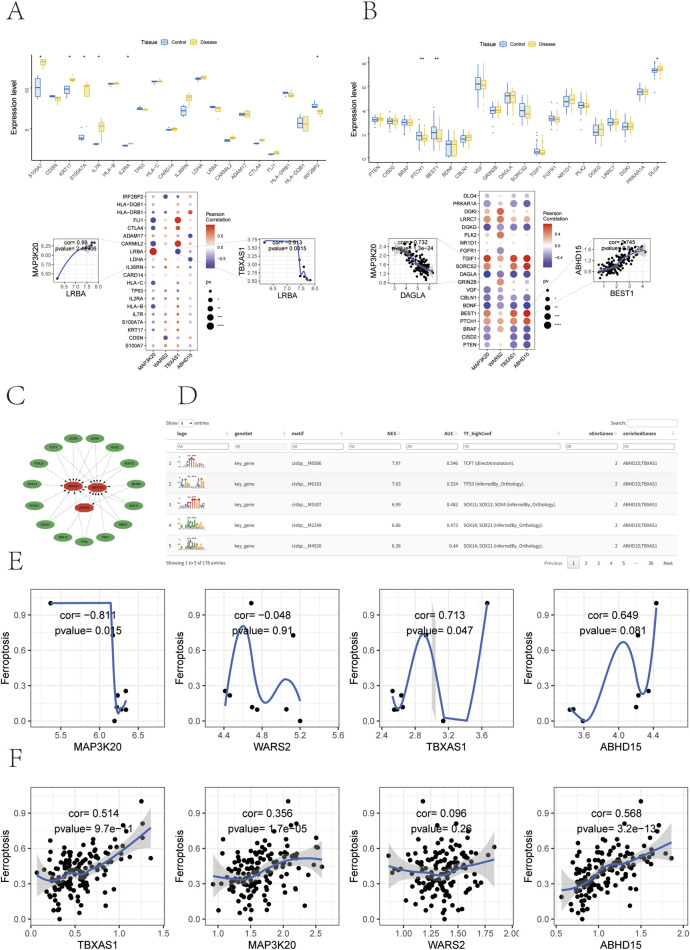
Expression of disease-related genes, transcriptional regulation, and their correlation with ferroptosis. **(A)** Top: Box plots showing expression levels of the top 20 psoriasis-related genes ranked by relevance score in Control and Disease groups. Bottom: Scatter plots (left, *MAP3K20* vs. *LRBA*; right, *TBXAS1* vs. *LRBA*) and heatmap depicting Pearson correlations between key Genes and *LRBA* in psoriasis. **(B)** Top: Box plots showing expression levels of the top 20 depression-related genes ranked by relevance score in Control and Disease groups. Bottom: Scatter plots (left, *MAP3K20* vs. *DAGLA*; right, *ABHD15* vs. *BEST1)* and heatmap depicting Pearson correlations between key Genes and *DAGLA/BEST1* in depression. **(C)** Network visualization of transcription factor (TF) motifs enriched in key Genes. **(D)** Table showing the top 5 enriched transcription factor (TF) motifs, displaying normalized enrichment scores (NES), area under the curve (AUC), and associated genes. **(E)** Correlation analysis between psoriasis key Genes and ferroptosis. **(F)** Correlation analysis between depression key Genes and ferroptosis.

In psoriasis, *MAP3K20* showed strong positive correlation with *LRBA* (r = 0.990), while *TBXAS1* demonstrated strong negative correlation with *LRBA* (r = −0.913) (Figure 6A). *LRBA* (LPS-responsive beige-like anchor protein) plays crucial roles in intracellular vesicle transport, immune cells activation, and inflammatory signal regulation, serving as a critical link between immune homeostasis and psoriasis. In depression, *ABHD15* exhibited significant positive correlation with *BEST1* (r = 0.745), while *MAP3K20* showed significant negative correlation with *DAGLA* (r = −0.732) (Figure 6B). The *BEST1* gene encodes bestrophin-1, a multifunctional membrane protein that primarily functions as a calcium-activated chloride channel. This gene, prominently expressed in ocular and brain tissues, participates in various physiological processes, and its dysfunction is associated with depression.

Using our key Genes as the gene set for analysis, we discovered their regulation by multiple transcription factors through common mechanisms. We performed enrichment analysis of these transcription factors using cumulative recovery curves. Motif-TF annotation and important gene selection analysis revealed that the motif sequence cisbp__M6506 showed the highest normalized enrichment score (NES: 7.97). Notably, ^SOX^ family transcription factors (*SOX11, SOX12, SOX4, SOX14, SOX21*) appeared repeatedly in the results. Previous studies have demonstrated the significant roles of the *SOX* family in inflammatory responses, immune regulation, skin diseases (including psoriasis), and neurological development and mental health (Rioux et al., 2020; Malhotra et al., 2013; Stevanovic et al., 2021; Baran et al., 2025). We presented the top 30 motifs enriched for key Genes and their corresponding transcription factors (Figures 6C,D).

### 3.7 Correlation between key genes and ferroptosis

Ferroptosis plays a crucial role in the development of psoriatic skin lesions through the regulation of oxidative stress, lipid peroxidation, and immune-inflammatory responses. These mechanisms generate inflammatory factors and metabolic alterations that can affect neurological function, thereby increasing the risk of depression in patients. To further investigate the relationship between key gene expression levels and ferroptotic molecular mechanisms, we obtained 484 ferroptosis-related genes from the FerrDb database (http://www.zhounan.org/ferrdb). We first quantified ferroptosis levels using the ssGSEA method and then assessed the correlation between key gene expression and ferroptosis levels through Pearson correlation analysis. Statistical analysis was performed using the cor.test() function, and correlations were visualized using scatter plots. In psoriasis, *MAP3K20* and *TBXAS1* showed significant correlation with ferroptosis levels (Figure 6E). In depression, *MAP3K20, TBXAS1,* and *ABHD15* demonstrated significant positive correlation with ferroptosis levels (Figure 6F).

### 3.8 Single-cell sequencing data analysis of psoriasis and depression

**FIGURE 7:**
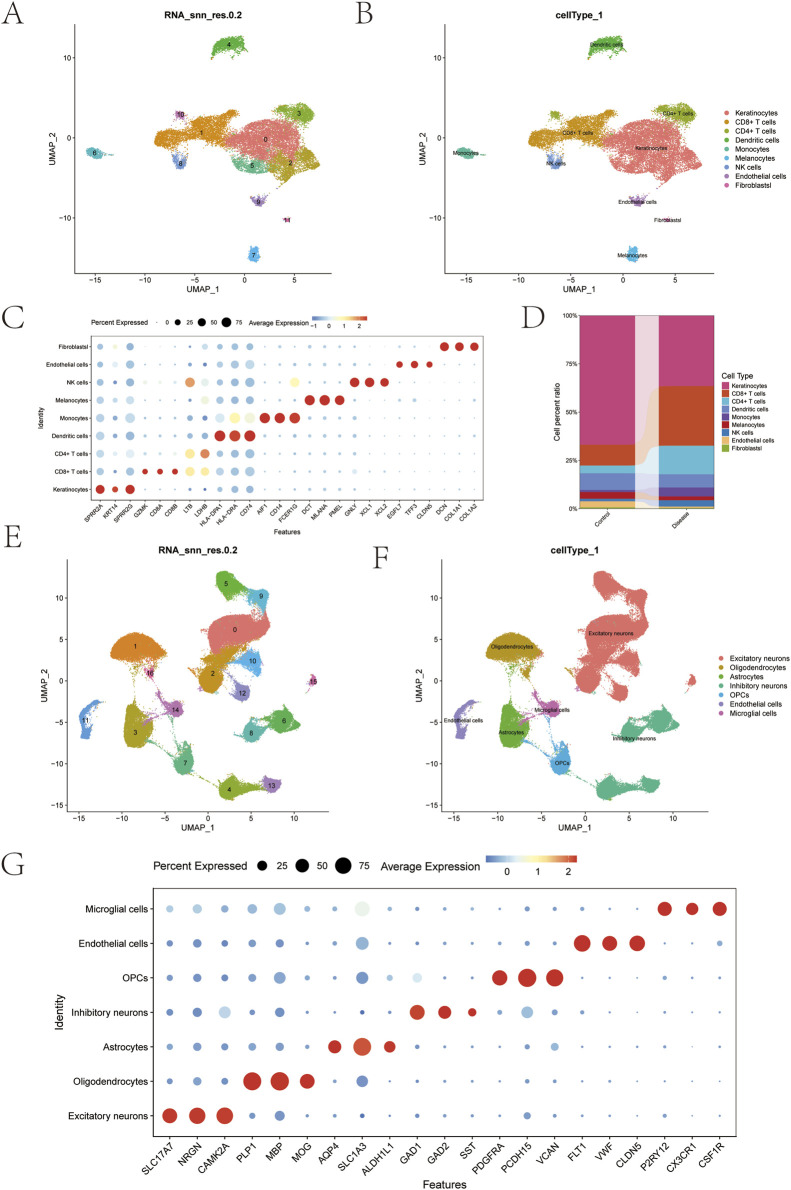
Single-cell clustering and cell type annotation in psoriasis and depression samples. **(A)** UMAP dimensionality reduction clustering plot of psoriasis single-cell sequencing data (12 cell clusters, clustering resolution RNA_snn_res.0.2). **(B)** Cell type annotation of 12 psoriasis cell clusters, identifying 9 cell types: keratinocytes, CD8^+^ T cells, CD4^+^ T cells, dendritic cells, monocytes, melanocytes, NK cells, endothelial cells, and fibroblasts. **(C)** Bubble plot showing expression patterns of classical markers across 9 cell types in psoriasis (dot size represents the proportion of cells expressing the gene, color indicates average gene expression level). **(D)** Stacked bar plot showing the proportion of cell types in Control and Disease groups of psoriasis samples. **(E)** UMAP dimensionality reduction clustering plot of depression single-cell sequencing data (17 cell clusters, clustering resolution RNA_snn_res.0.2). **(F)** Cell type annotation of 17 depression cell clusters, identifying 7 cell types: excitatory neurons, oligodendrocytes, astrocytes, inhibitory neurons, oligodendrocyte precursor cells (OPCs), endothelial cells, and microglia. **(G)** Bubble plot showing expression patterns of classical markers across 7 cell types in depression (dot size represents the proportion of cells expressing the gene, color indicates average gene expression level).

This study utilized the psoriasis dataset GSE220116 and depression dataset GSE213982. Gene expression profile data were initially imported using the Seurat package. Based on comprehensive quality assessment of multiple samples, we established filtering criteria: removing detected outliers and low-quality cells containing fewer than 200 genes. Subsequently, we employed the DoubletFinder package to eliminate doublets. Ultimately, 24,672 high-quality cells were retained for subsequent analysis, with post-filtering data quality demonstrated through violin plots and scatter plots (Supplementary Figures S2A,B). We identified 2,000 highly variable genes (Supplementary Figure S2C). The data underwent sequential standardization, normalization, principal component analysis (PCA), and Harmony batch correction analysis (Supplementary Figures S2D–F). Following dimensionality reduction using the Uniform Manifold Approximation and Projection (UMAP) algorithm, all cells were classified into 12 subgroups (Figure 7A). Through annotation using known cell markers, we ultimately identified 9 major cell types: Keratinocytes, CD8^+^ T cells, CD4^+^ T cells, Dendritic cells, Monocytes, Melanocytes, NK cells, Endothelial cells, and Fibroblasts. Figure 7B and C present bubble plots showing the expression of classical markers for these nine cell types, while Figure 7D displays a bar chart of cell proportions under different grouping conditions.

Similarly, after filtering the depression dataset, 126,555 high-quality cells were retained for subsequent analysis (Supplementary Figures S3A,B). The 2,000 highly variable genes were visualized using volcano plots (Supplementary Figure S3C). Subsequently, the data underwent standardization, normalization, PCA dimensionality reduction, and Harmony batch effect correction (Supplementary Figures S3D–F). UMAP dimensionality reduction analysis classified the cells into 17 subgroups (Figure 7E). Based on the expression patterns of typical marker genes, these subgroups were annotated into 7 cell types: excitatory neurons, oligodendrocytes, astrocytes, inhibitory neurons, OPCs, endothelial cells, and microglial cells (Figure 7F). Additionally, a bubble plot displaying the classical markers for these cell types was generated (Figure 7G).

### 3.9 Single-cell expression analysis of key genes and disease gene co-expression network analysis

**FIGURE 8:**
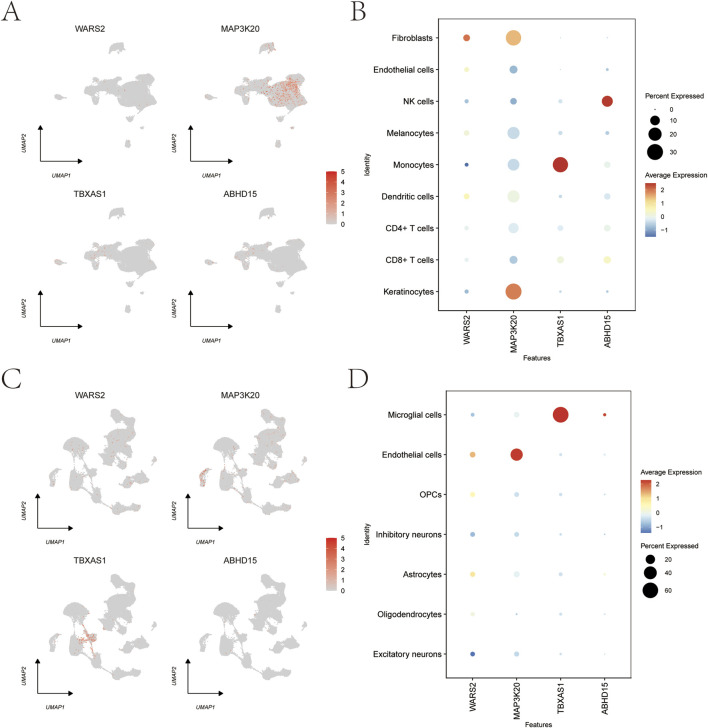
Single-cell expression patterns of key Genes in psoriasis and depression visualized by UMAP-based FeaturePlot. **(A)** Expression distribution of *WARS2, MAP3K20, TBXAS1*, and *ABHD15* in the single-cell transcriptome data of psoriasis. **(B)** Dot plot showing the average expression levels of *WARS2, MAP3K20, TBXAS1,* and *ABHD15* across different cell types in psoriasis. High expression patterns were observed for *WARS2* in fibroblasts, *MAP3K20* in keratinocytes, *TBXAS1* in monocytes, and *ABHD15* in NK cells. **(C)** Expression distribution of *WARS2, MAP3K20, TBXAS1*, and *ABHD15* in the single-cell transcriptome data of depression. **(D)** Dot plot depicting the average expression levels of the aforementioned genes across different cell types in depression. Key expression patterns include high expression of *WARS2* and *MAP3K20* in endothelial cells, and *TBXAS1* and *ABHD15* in microglial cells.

We analyzed the expression patterns of key Genes at single-cell resolution using the Dotplot and FeaturePlot functions from the SeuratR package to visualize their expression across different cell types in psoriasis and depression. In the psoriasis single-cell dataset, *WARS2* showed high expression in fibroblasts, *MAP3K20* was highly expressed in keratinocytes, *TBXAS1* showed elevated expression in monocytes, and ABHD15 was predominantly expressed in NK cells (Figures 8A,B). In the depression single-cell dataset, both *WARS2* and *MAP3K20* exhibited high expression levels in endothelial cells, while *TBXAS1* and *ABHD15* showed elevated expression in microglial cells (Figures 8C,D).

We selected the top 20 psoriasis-related genes ranked by Relevance score from the GeneCards database and analyzed their co-expression networks with key Genes (Supplementary Figures S4A–H). *ABHD15* showed strong negative correlation with *S100A7A* (R = −0.74), a core inflammatory gene involved in skin lesion inflammation. *MAP3K20* exhibited strong negative correlation with *IL2RA* (R = −0.61), an immune regulatory gene associated with psoriasis susceptibility. *TBXAS1* demonstrated moderate negative correlation with *CDSN* (R = −0.44), a skin barrier gene involved in skin pathology. These co-expression patterns highlight the close relationship between key Genes and psoriasis immune and skin pathology.

Similarly, we analyzed co-expression networks between key Genes and the top 20 depression-related genes (Supplementary Figures S5A–H). The key Genes *WARS2, MAP3K20, TBXAS1*, and *ABHD15* showed significant co-expression associations with depression-related genes, including BDNF (brain-derived neurotrophic factor, a core depression-related gene regulating neuroplasticity) and *CISD2* (a depression-associated gene involved in cellular iron homeostasis and oxidative stress). Specifically, *WARS2* showed strong negative correlation with *BDNF* (R = −0.74) and significant positive correlation with *BRAF* (R = 0.53). *MAP3K20* exhibited strong negative correlations with both *BDNF* (R = −0.73) and *CISD2* (R = −0.73). *TBXAS1* demonstrated the strongest negative correlation with *BDNF* (R = −0.77) and strong negative correlation with *CISD2* (R = −0.7). *ABHD15* showed negative correlations with both *BDNF* (R = −0.62) and *CISD2* (R = −0.42).

Key Genes including *ABHD15, MAP3K20*, and *TBXAS1* exhibited significant co-expression associations with core functional genes in both psoriasis and depression. These associations reflect their molecular regulatory roles in different disease pathological processes, with particularly prominent negative correlation patterns, providing insights for cross-disease molecular mechanism research.

### 3.10 Therapeutic drug prediction analysis for key genes

**FIGURE 9:**
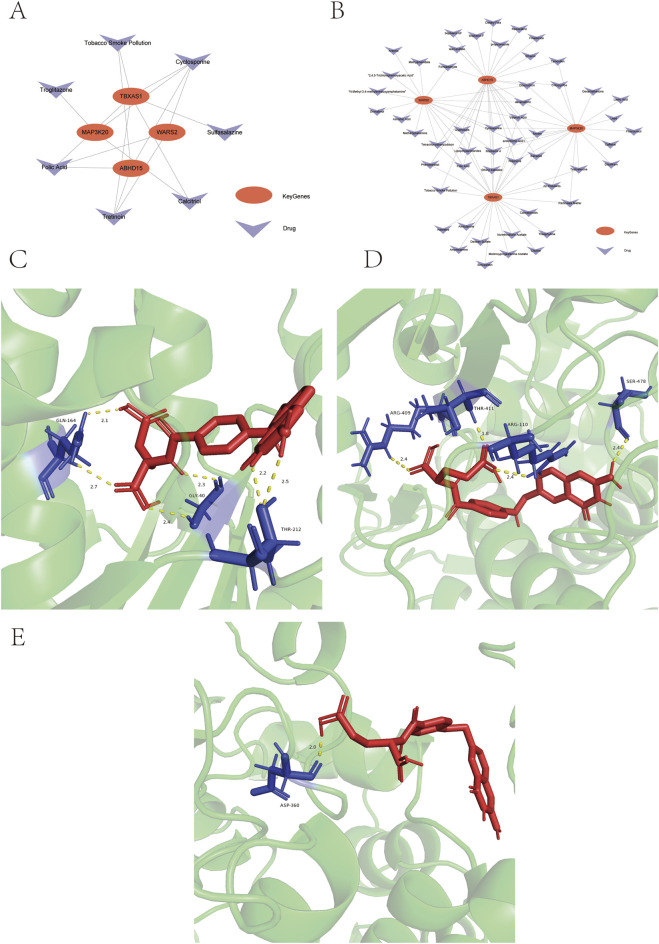
Drug-gene interaction networks and molecular docking results. **(A)** Drug-gene interaction network from CTD analysis in psoriasis (red ellipses: key Genes, blue: drugs). **(B)** Drug-gene interaction network from CTD analysis visualized by Cytoscape in depression (red ellipses: key Genes, blue: drugs). **(C–E)** Molecular docking of folic acid with *WARS2* (**(C)**, binding energy −9.0 kcal/mol), *TBXAS1* (**(D)**, binding energy −8.0 kcal/mol), and *ABHD15* (**(E)**, binding energy −7.4 kcal/mol). Red sticks represent folic acid, blue sticks represent protein interacting residues, and yellow dashed lines indicate hydrogen bonds.

The Comparative Toxicogenomics Database (CTD) was utilized to analyze potential drug interactions with four key Genes (*MAP3K20, WARS2, TBXAS1*, and *ABHD15*). Through CTD analysis, we identified that in psoriasis, three drugs interacted with *MAP3K20* and *WARS2* (Figure 9A), six drugs interacted with *TBXAS1*, and four drugs interacted with *ABHD15*. In depression, twenty drugs showed interactions with *MAP3K20*, seventeen drugs with *WARS2*, and twenty-seven drugs with both *TBXAS1* and *ABHD15*. Notably, Folic Acid demonstrated interactions with *WARS2, TBXAS1*, and *ABHD15* in both diseases. The predicted drug-target interactions were visualized using Cytoscape (Figure 9B). For molecular docking analysis, we selected the following protein-compound pairs: *WARS2*:Q9UGM6-Folic Acid, *TBXAS1*:P24557-Folic Acid, and *ABHD15*:Q6UXT9-Folic Acid. The docking binding energies were presented in tabular form and the docking results were visualized. Molecular docking results revealed binding energies of −9.0 kcal/mol for *WARS2*:Q9UGM6-Folic Acid (Figure 9C), −8.0 kcal/mol for *TBXAS1*:P24557-Folic Acid (Figure 9D), and −7.4 kcal/mol for *ABHD15*:Q6UXT9-Folic Acid (Figure 9E).

## 4 Discussion

Psoriasis, affecting 2%–3% globally, and major depressive disorder (MDD), with 10%–15% lifetime prevalence, demonstrate significant comorbidity. Depression exacerbates psoriasis through neuroimmune mechanisms and reduces treatment adherence, while psoriatic inflammation releases pro-inflammatory mediators (TNF- α, IL-6) that disrupt neurochemical balance, creating a bidirectional pathophysiological relationship that severely impacts quality of life (Depression WHO, 2017; Parisi et al., 2017; Sahi et al., 2020; Dregan et al., 2019; Jensen et al., 2016; Wu et al., 2017; Maqbool et al., 2021; Mrowietz et al., 2023; Keenan and Granstein, 2025; Li et al., 2024). Multidisciplinary care is essential for psoriasis management. Current guidelines recommend integrating psychological assessment with systemic therapy (biologics, small molecules) to reduce comorbidity burden (Elmets et al., 2019). SSRIs are first-line antidepressants for comorbid depression, though dermatology-psychiatry collaboration is needed to monitor potential cutaneous adverse effects (Kenkare et al., 2025). Depression screening is emphasized for patients with severe lesions or impaired quality of life (Daudén et al., 2013). Early identification and intervention are crucial. Clinicians should systematically assess depression risk in psoriasis patients, particularly those with severe lesions, persistent pruritus, or poor treatment response. Multi-omics integration and analysis of shared pathways and key genes (inflammatory and neuroimmune nodes) can elucidate comorbidity mechanisms, identify biomarkers, and enable precision interventions to improve both cutaneous and emotional outcomes.

Immune dysregulation drives psoriasis-depression comorbidity. Psoriasis inv olves Th1/Th17 axis activation, with IL-23-IL-17 and TNF-a pathways mediatin g epidermal hyperproliferation and inflammation (Ghoreschi et al., 2021; Thaçi et al., 2015). In depression, peripheral pro-inflammatory cytokines (TNF- α, IL-6) activate central microglia and astro cytes, disrupting monoaminergic neurotransmission and producing inflammation-r elated depressive phenotypes (Miller and Raison, 2016). In psoriasis-depression comorbidity, peripheral i nflammatory signals (TNF- α, IL-6) and stress axis dysregulation create bidirect ional amplification: inflammation drives central responses and depressive behaviors, while HPA axis dysfunction and aberrant cutaneous glucocorticoid signaling exacerbate peripheral inflammation, revealing shared inflammatory pathology (Dantzer et al., 2008; Slominski et al., 2006).

We identified four key genes *MAP3K20, WARS2, TBXAS1, ABHD15* as molecular hubs in psoriasis-depression comorbidity, spanning immune, metabolic, and stress pathways. In psoriasis, these genes enriched in FoxO/IL-17/ErbB signaling (*MAP3K20*), IL-17/proteasome/DNA sensing (*WARS2*), DNA replication/proteasome (*TBXAS1*), and relaxin/HIF-1 signaling (*ABHD15*). In depression, they enriched in lipid metabolism (*MAP3K20*), synaptic vesicle cycling (*WARS2*), complement cascades (*TBXAS1*), and TGF- β/NF- κ B/Notch pathways (*ABHD15*). Immune profiling revealed shared CD8^+^ T cell, dendritic cell, and neutrophil infiltration, suggesting common pathological mechanisms and potential therapeutic targets.

The Key Genes identified in this study *MAP3K20, WARS2, TBXAS1*, and *ABHD15* may be closely associated with the pathological process of psoriasis-depression comorbidity. *MAP3K20*, a member of the MAPKKK family, is a serine/threonine protein kinase crucial in cellular stress responses. As an upstream regulator of MAPK signaling, it primarily activates JNK and p38 cascades, participating in cell apoptosis, inflammatory responses, and cell cycle regulation (Obata et al., 2000). MAPK signaling pathways, especially JNK and p38, play important roles in psoriasis pathogenesis, regulating keratinocyte proliferation and inflammation while mediating activation and cytokine secretion of T cells and dendritic cells (Guo et al., 2023; Sakurai et al., 2019; Furue et al., 2020). Literature suggests potential roles for *MAP3K20* and MAPK signaling in depression (Li et al., 2023). Our study found that in psoriasis, *MAP3K20* directly interacts with key immune and metabolism-related genes such as IL2RA and *LDHA*, participating in T cell activation and immune cell metabolism regulation. In depression, *MAP3K20* shows significant correlation with the core molecule *BDNF*, indicating important associations with both disease pathological processes.

*WARS2* gene encodes mitochondrial tryptophanyl-tRNA synthetase, essential for mitochondrial protein synthesis. While direct associations between *WARS2* and psoriasis or depression have not been previously reported, studies have shown that *WARS2* mutations can lead to various neurological disorders, including infantile-onset leukoencephalopathy and parkinsonian symptoms (Theisen et al., 2017; Maffezzini et al., 2019). Our study revealed pathway-specific regulatory patterns of *WARS2* in both diseases: in psoriasis, it is significantly enriched in IL-17 signaling (core pathway mediating skin inflammation), proteasome pathway (maintaining cellular protein homeostasis), and cytosolic DNA sensing pathway (regulating immune response activation); in depression, it is enriched in synaptic vesicle cycling (affecting neural signal transmission efficiency), insulin secretion (linking metabolic-neural regulatory interactions), and complement-coagulation cascade pathways (participating in neuroinflammatory microenvironment formation).

*TBXAS1* gene encodes a key microsomal enzyme catalyzing the conversion of prostaglandin H2 (PGH2) to thromboxane A2 (TXA2). Studies have found that TXA2 can promote IL-17A production by Vγ4+ γδ T cells, driving psoriasis-like dermatitis in mice (Ueharaguchi et al., 2018). Research on this gene in depression is limited. Our GSEA revealed TBXAS1 enrichment in DNA replication, proteasome, and RNA polymerase pathways in psoriasis, and in TGF-β, NF-κB, and Notch signaling pathways in depression. CIBERSORT immune infiltration analysis showed significant positive correlations between TBXAS1 and CD8^+^ T cells and M2 macrophages in psoriasis, while in depression, it positively correlates with M1 and M2 macrophages and negatively correlates with M0 macrophages and resting CD4^+^ memory T cells.

*ABHD15* is an important lipid metabolism regulator belonging to the α/β-hydrolase superfamily. While direct studies linking *ABHD15* to psoriasis and depression are lacking, research suggests its potential roles in neuroinflammation and neuroimmune responses (Xia et al., 2018; Gabbia and De Martin, 2023). Our study found *ABHD15* enrichment in relaxin signaling, HIF-1 signaling, and glucagon signaling pathways in psoriasis, and in cholesterol metabolism, β-alanine metabolism, and histidine metabolism pathways in depression.

Ferroptosis, a regulated cell death form, participates in both diseases: elevated *PTGS2* (a ferroptosis marker) in psoriasis and its neuroinflammatory role in depression suggest therapeutic potential (Xiang et al., 2025; Tao et al., 2025; Liang et al., 2025; Peng et al., 2025; Wang et al., 2023). Single-cell transcriptome analysis identified distinct cell type compositions in psoriasis and depression samples. CTD prediction and molecular docking confirmed folic acid as a common drug targeting three of the four genes, with stable binding affinity.

Study limitations include inadequate sample representativeness and single SNP-dependent instrumental variables. Future research should conduct cross-ethnic multicenter validations, GWAS meta-analyses, and integrate pQTL/eQTL/meQTL for refined causal inference.

## 5 Conclusion

Through multi-omics integration analysis, this study systematically elucidated the molecular mechanisms of psoriasis-depression comorbidity, identified four key biomarkers (*MAP3K20, WARS2, TBXAS1*, and *ABHD15*), discovered that inflammatory dysregulation and metabolic disorders form the core pathological basis, demonstrated that immune microenvironment abnormalities drive disease progression, and proposed folic acid as a potential therapeutic target, providing new insights for clinical translation.

## Data Availability

The datasets presented in this study can be found in online repositories. The names of the repository/repositories and accession number(s) can be found in the article.
