# Supplementary figures and images for: Molecular mechanisms underlying psoriasis and depression: an integrated analysis using mendelian randomization, transcriptomics, and single-cell sequencing
Source: Front Mol Med. 2026 Apr 15;6:1770665. doi: 10.3389/fmmed.2026.1770665 (PMC13124126; doi:10.3389/fmmed.2026.1770665)

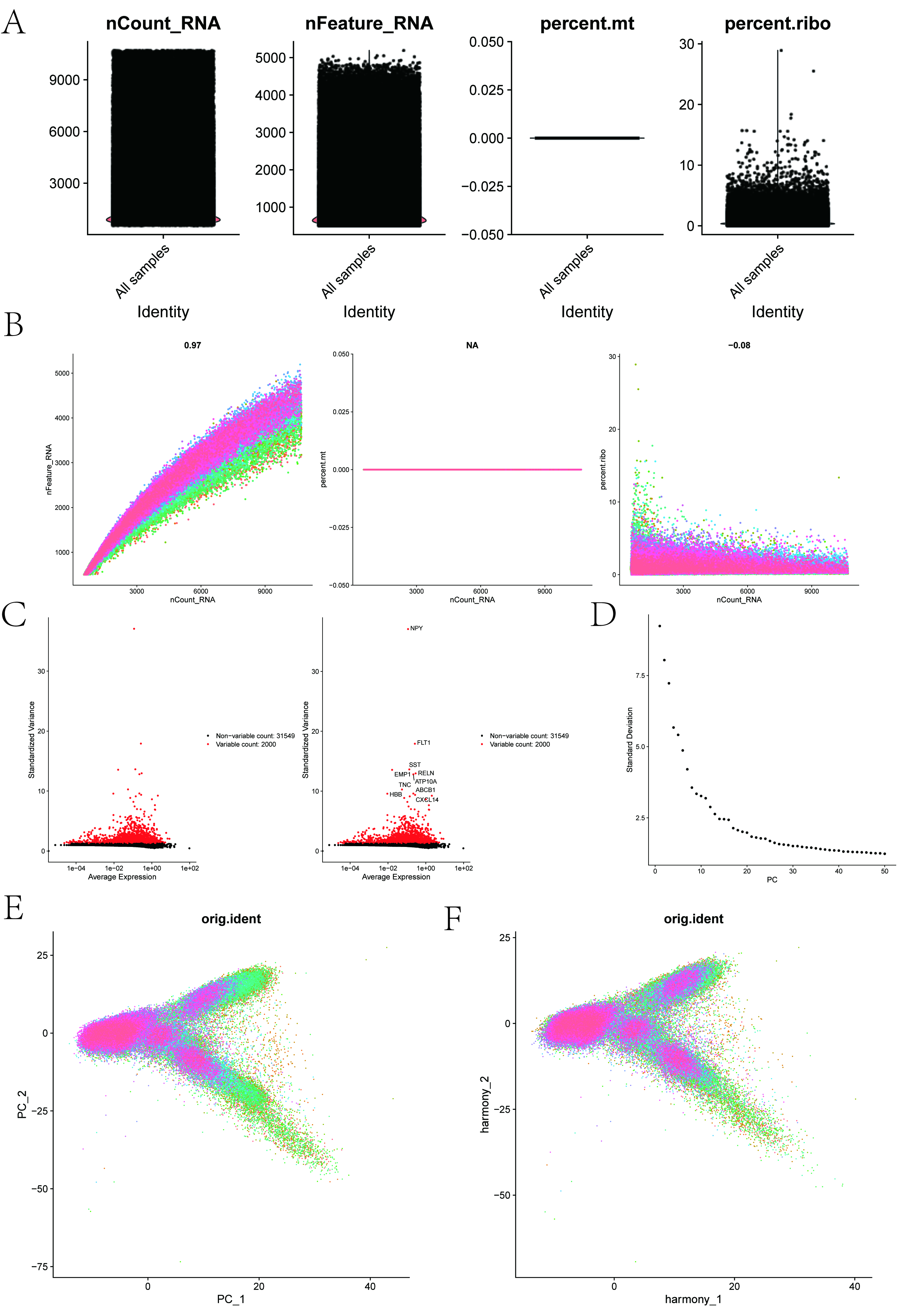

Supplement: Supplementary file 2 [file Image3.tif]

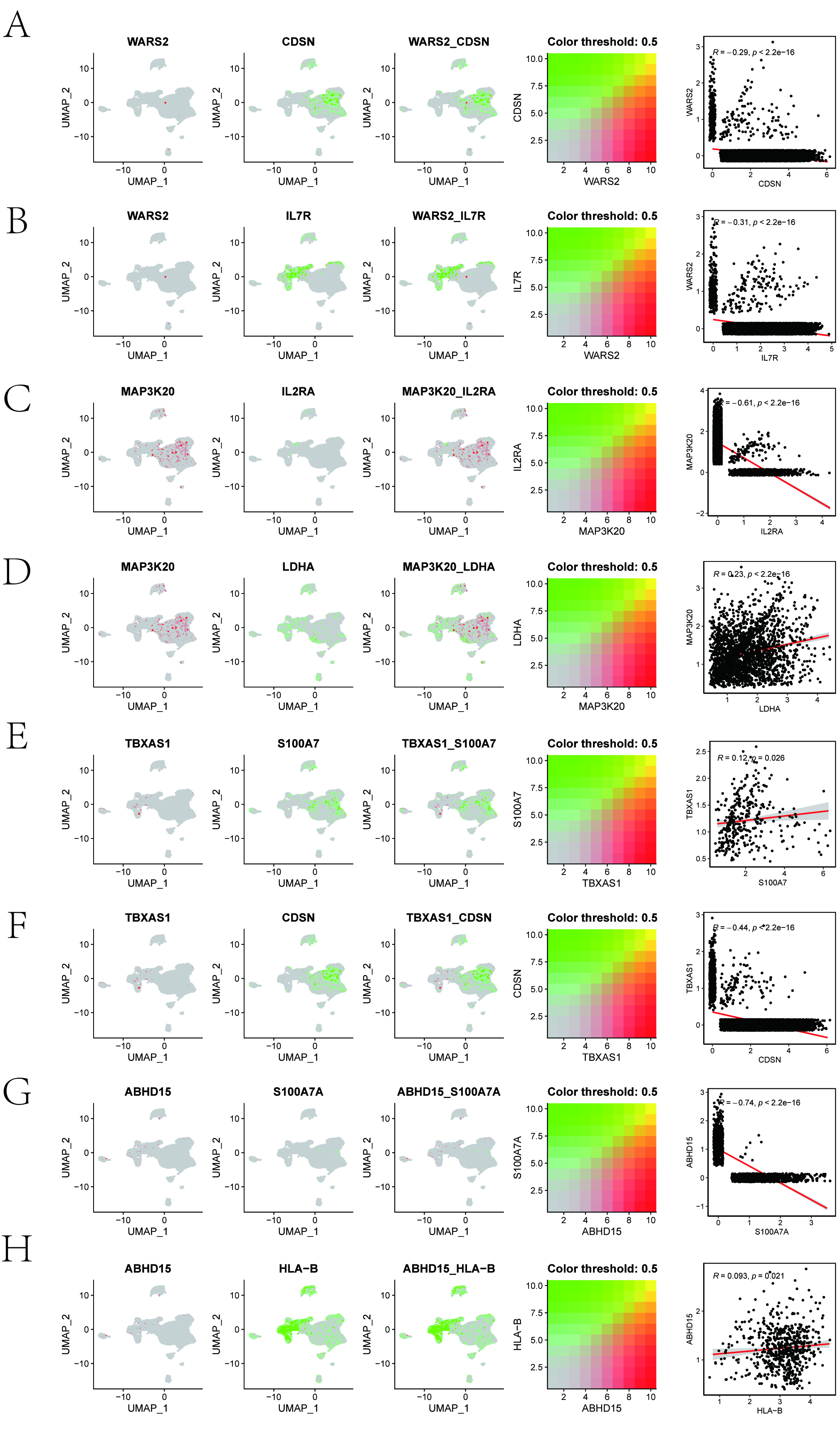

Supplement: Supplementary file 3 [file Image4.tif]

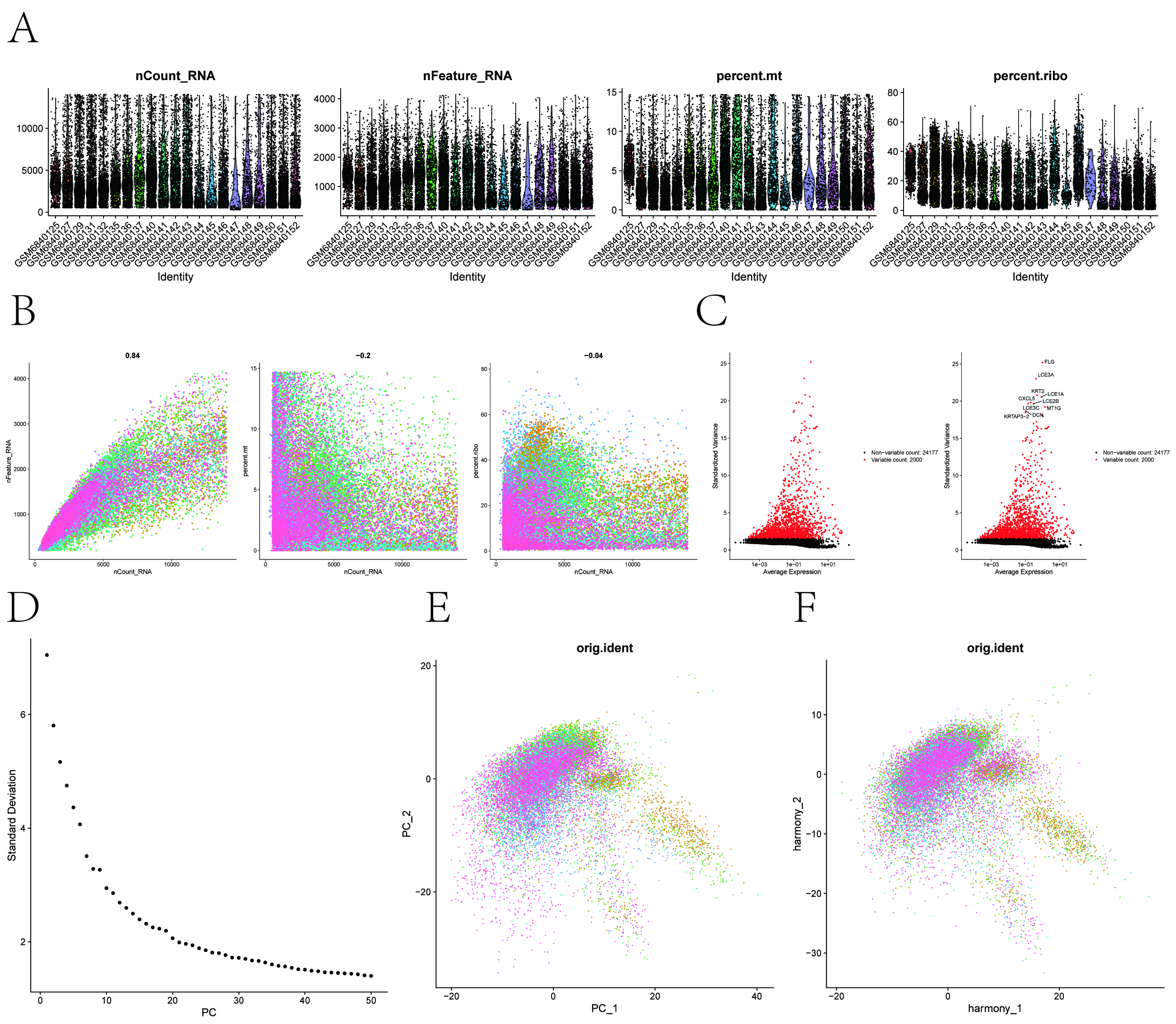

Supplement: Supplementary file 4 [file Image2.tif]

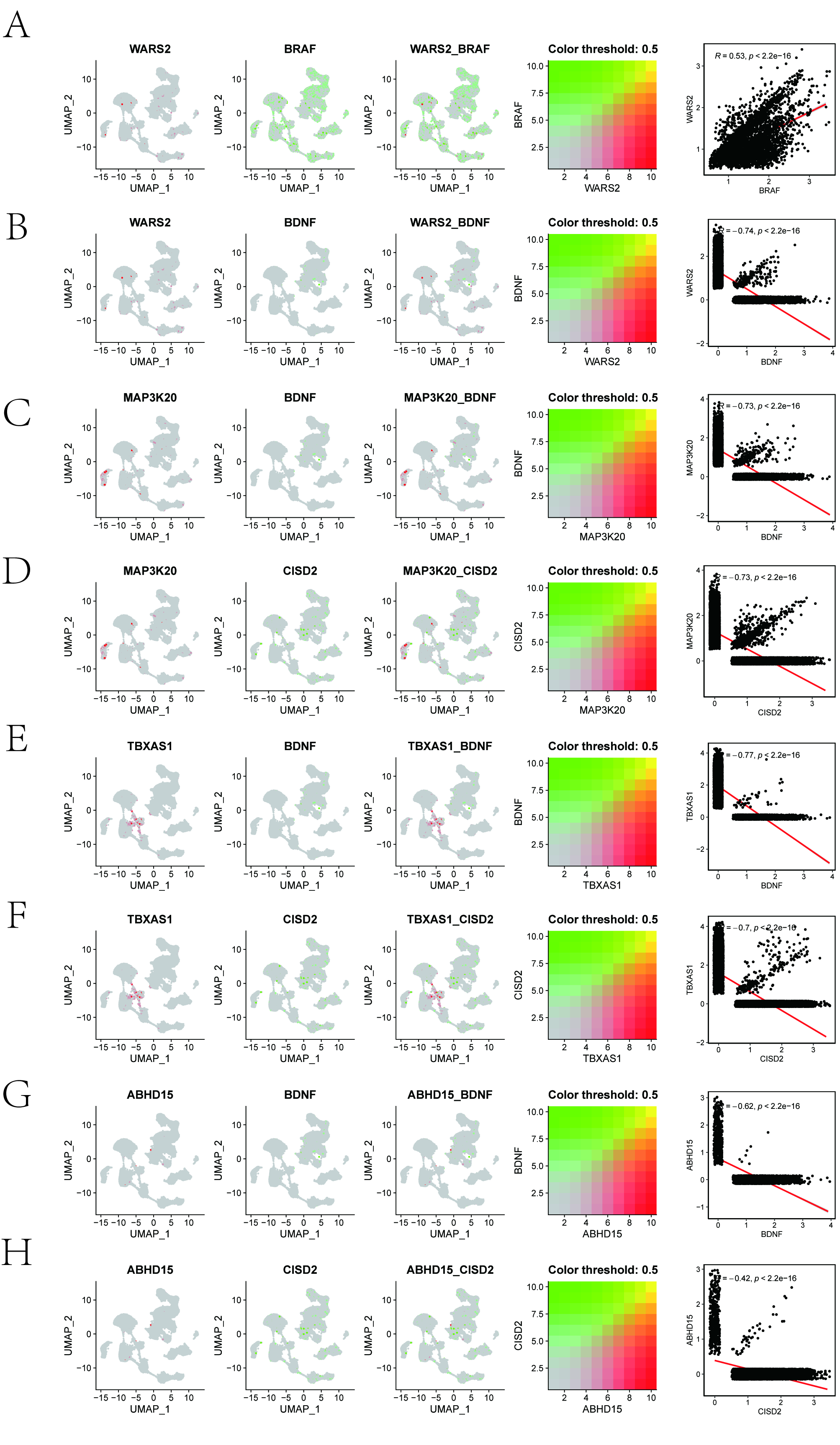

Supplement: Supplementary file 5 [file Image5.tif]
